# Supplementary material for: Predicting compound activity from phenotypic profiles and chemical structures
Source: Nat Commun. 2023 Apr 8;14:1967. doi: 10.1038/s41467-023-37570-1 (PMC10082762; doi:10.1038/s41467-023-37570-1)
Supplement: Supplementary file 1 — Supplementary Information [file 41467_2023_37570_MOESM1_ESM.pdf]

Supplementary Material

Experimental design

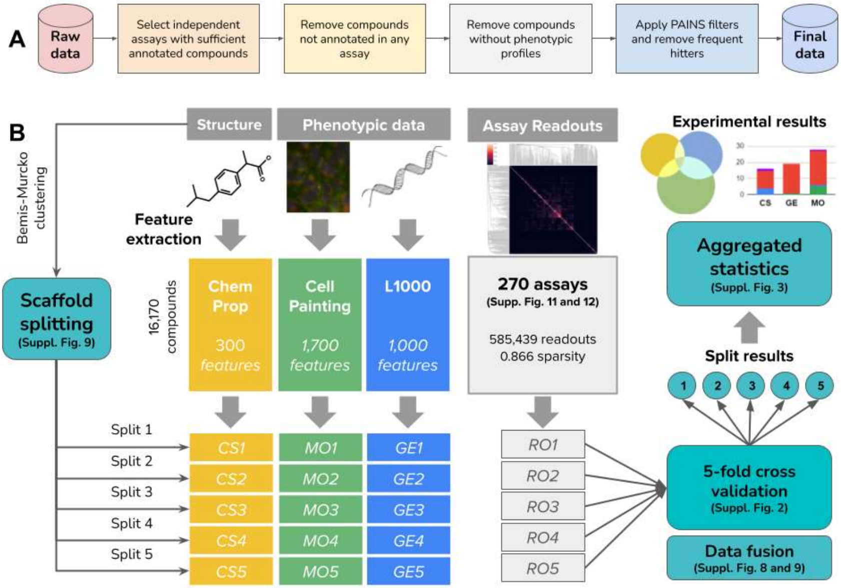

Supplementary Figure 1. Illustration of the experimental design in this study. A) Data selection and filtering pipeline to construct the dataset used in this study. The process is linear and the order of steps is followed one at a time. We first select 270 assays from more than 500 available (see Supplementary Figure 12 and 13), and with those targets fixed, we proceed to clean the list of compounds with various other filters. B) We considered the problem of assay prediction from three compound representations: features of the chemical structure, and phenotypic features of the effect of compounds measured by imaging (Cell Painting) and gene expression (L1000). We conducted a 5-fold cross-validation experiment splitting the compounds in 5 groups according to scaffold similarity using the Bemis-Murcko clustering. The profiles for compounds in each of these groups were separated together with the corresponding assay readouts. The training of models and test of predictions is carried out independently for each fold, and the results are aggregated to generate summarized statistics of the experimental results.

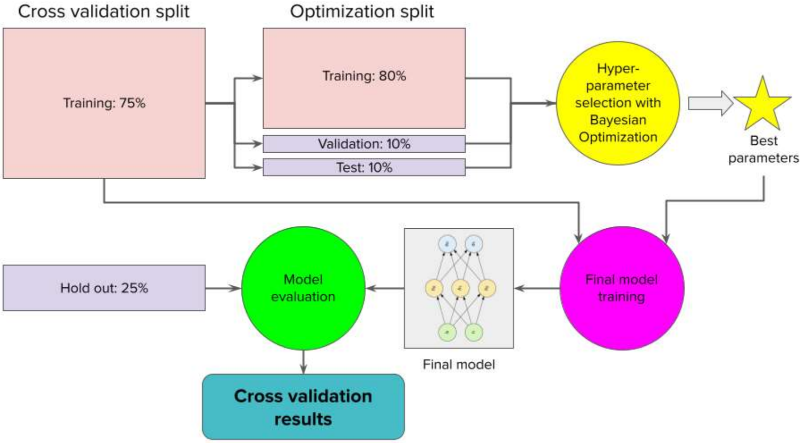

Supplementary Figure 2. Pipeline of cross-validation experiments. The models trained and evaluated in our experiments are conducted following this protocol: for each split in the 5-fold validation scheme, we take the training dataset and split it again in three parts: 80% for training, 10% for validation and 10% for testing. In this partition, we run hyperparameter search using Bayesian optimization to calibrate the parameters described in the Methods section, subsection Predictive model and data fusion. The Bayesian optimization model uses the 10% assigned for validation to search better parameters at each iteration, and when the search is complete, a final evaluation is performed on the 10% test set with a subset of the best candidates to identify the hyperparameters with better out of sample generalization. These best hyperparameters are used to train a final model with the entire training data in the original split, which is later evaluated with the subset held out for test. The results out of this evaluation are reported in the main text as well as in the rest of the manuscript.

Additional results

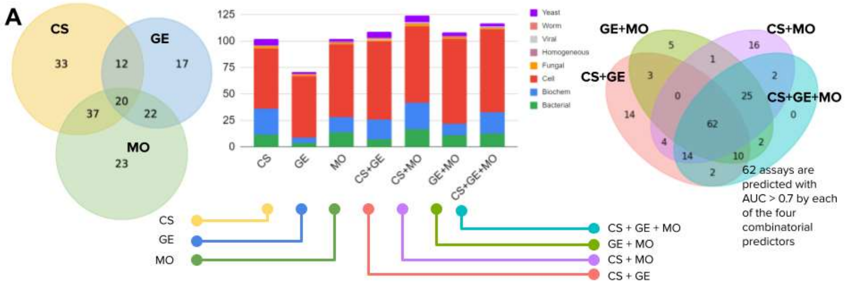

B

	CS	GE	MO	CS+GE	CS+MO	GE+MO	CS+GE+MO
**Mean AUC**	0.630	0.592	0.637	0.645	0.661	0.652	0.665
**Mean AUC > 0.7**	98	63	100	97	115	106	111
**Median AUC > 0.7**	102	71	102	109	124	108	117
**Retrospective ★**	-	-	-	151	157	139	174

Supplementary Figure 3. Assays predicted with AUROC > 0.7. Summary of the number of assays predicted with models that have AUROC > 0.7, which is a lower performance threshold than the one used throughout our study. The total number of acceptable assay predictors increases when the threshold is lower, and chemical structures can yield more predictors that meet this level of performance. Importantly, predictors that reach performance above 0.7 AUROC are also capable of improving hit rates in many cases (see yellow points in Supplementary Figure 7). The row “Retrospective” in Table B presents the number of assays with AUROC > 0.7 that would be predicted by any of the modalities individually or their combinations.

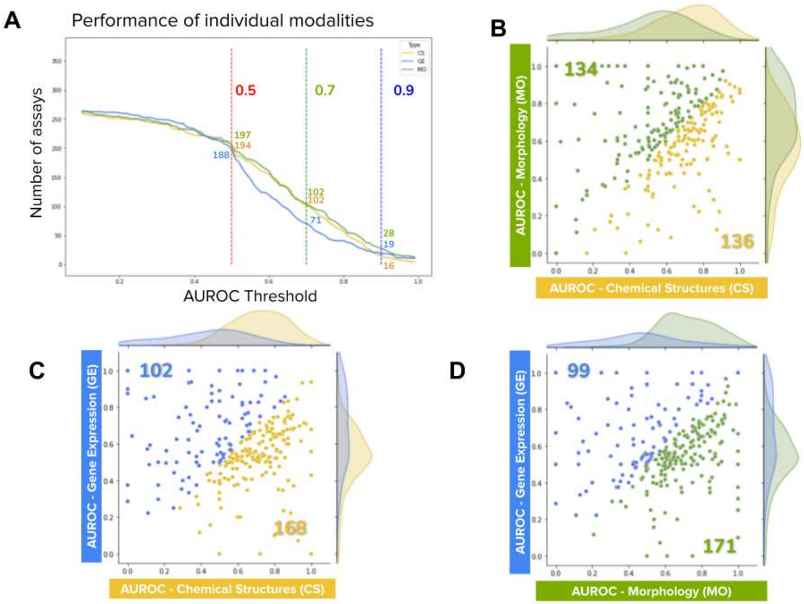

Supplementary Figure 4. Comparison of performance of individual modalities. Area under the curve (AUROC) performance of the three individual modalities evaluated in our study: Chemical Structures (CS), Gene Expression (GE), and Morphology (MO). A) Number of assays predicted by each modality at specific AUROC thresholds. As the AUROC threshold is increased, the number of assays meeting the threshold decreases for all modalities. The two thresholds discussed in this paper are highlighted in green (0.7) and blue (0.9). B, C, D) Scatter plots of AUROC for pairs of modalities. Each point in the plots represents an assay, the x coordinate indicates the AUROC obtained in one modality, and the y axis represents the AUROC obtained in the other modality. Colors represent the three individual modalities: CS (yellow), GE (blue) and MO (green). Points (assays) above or below the diagonal (equal performance) are colored according to the modality that has the highest AUROC. The two colored numbers inside the plot indicate the total number of assays with higher AUROC with respect to the other modality in the same plot. The counts of points indicate the number of assays where one modality is better than the other. Note that there are many points far off the diagonal, indicating high AUROC in one modality but low in the other. This indicates potential for complementary and fusion among the different data modalities.

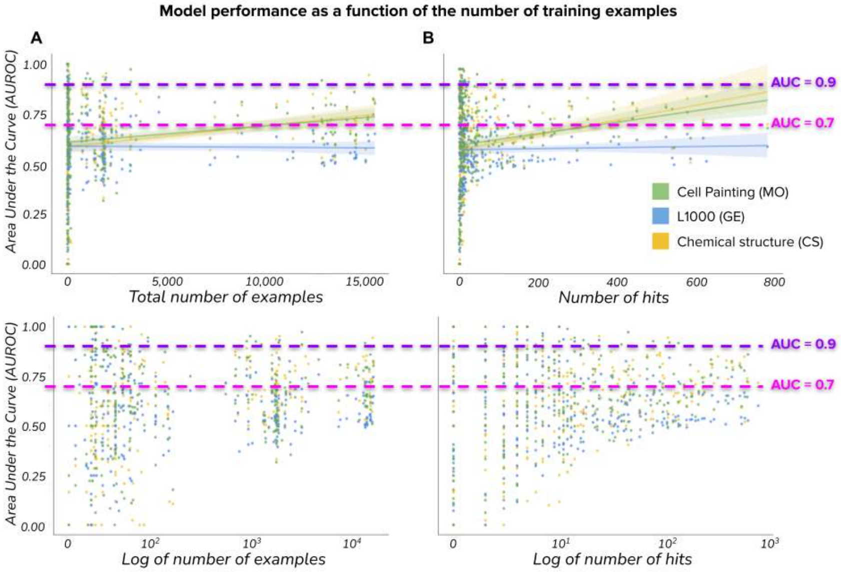

Supplementary Figure 5. Model performance as a function of the number of training examples. The performance of predictive models is slightly correlated with the number of available training examples; several assays can be predicted with high accuracy (AUROC > 0.9) using only a few example hits (points above the purple line). The plots show on the vertical axis the test set accuracy as a function of (A) the total number of example readouts, and (B) the number of hits available for training. Plots in the bottom row show the same data with log scale in the horizontal axis to highlight the trend with few examples. Lines in the plot in the top are linear regression with 95% confidence interval (colored regions). Each point is an assay predictor and its color indicates what data modality was used for training it. Note that assay prediction accuracy can vary from very low to very high with a small number of training examples, indicating that performance depends on the specific activity measured by the assay.

Scaffold-based splits — without balancing						
	MO	MO-BC	GE	GE-S	CS-GC	CS-MF
Mean AUPRC	0.261	0.252	0.234	0.231	0.232	0.223
Mean AUROC	0.657	0.637	0.592	0.587	0.630	0.610
AUC > 0.5	160.0	151.4	139.2	138.8	150.2	146.8
AUC > 0.7	91.2	83.2	57.2	59.4	88.4	81.6
AUC > 0.9	27.0	28.0	21.8	18.4	21.6	21.0
**Scaffold-based splits — with balancing**						
	MO	MO-BC	GE	GE-S	CS-GC	CS-MF
Mean AUPRC	0.251	0.246	0.228	0.225	0.233	0.222
Mean AUROC	0.654	0.626	0.589	0.582	0.631	0.608
AUC > 0.5	158.0	147.6	136.0	135.8	149.6	146.6
AUC > 0.7	91.2	80.4	59.4	58.0	87.6	80.8
AUC > 0.9	25.8	25.4	20.2	17.8	22.6	19.8

Average number of tested assays: **202.2**

Supplementary Table 1. 5-fold scaffold-based cross-validation results. The tables present the mean results of 5-fold scaffold-based cross-validation (see Supplementary Figure 10 and Supplementary Table 3) experiments according to usage of ChemProp's built-in balancing, which is performed molecule-wise. For each data modality, we used two encoding versions as follows: MO: original features and batch corrected (BC) features. GE: original features and scaled (S) or renormalized features using the L1 norm. CS: graph convolutional (GC) features and Morgan fingerprints (MF) (see also Supplementary Table 2).

Scaffold-based splits		
	CS-GC Multi-task	CS-GC Single-task
Mean PR-AUC	0.232	0.178
Mean AUC	0.630	0.525
AUC > 0.5	150.2	108.0
AUC > 0.7	88.4	44.8
AUC > 0.9	21.6	11.8

Supplementary Table 2. Comparison of single-task and multi-task training. Evaluation of predictors based on learned representations (graph convolutions) of chemical structures under two training regines: multi-task and single-task training. The results are the mean of 5-fold cross-validation experiments. The multi-task approach is trained to predict all available assays simultaneously, resulting in a single model trained end-to-end. The single-task approach trains one model per assay, resulting in 270 different predictors in our experiments. The results show that learning all predictors in a multi-task setting leverages the synergistic effect of using more data, and also simplifies the implementation and experimental workflow with a single model.

Enrichment factors

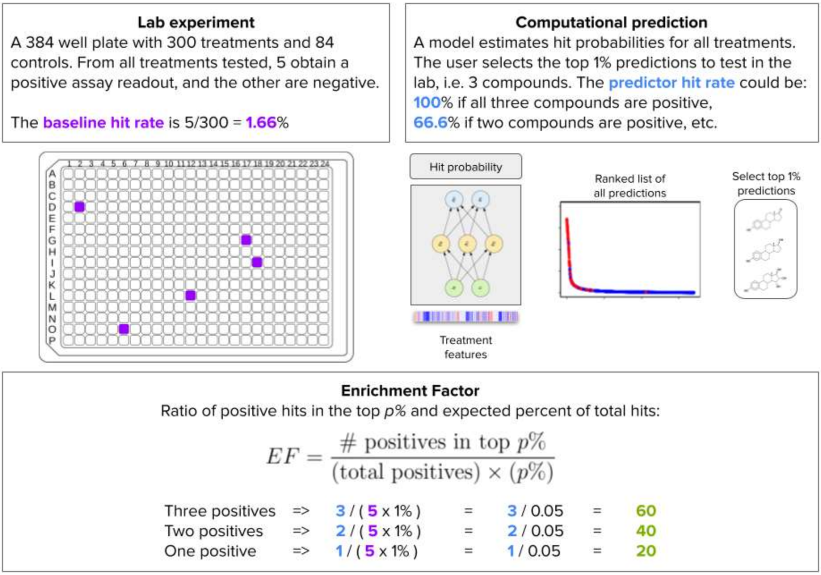

Supplementary Figure 6. Illustration of the "Folds of improvement" metric. The example assumes a chemist testing a set of 300 candidate compounds where only 5 of them are positive hits. The ratio of hits vs tested compounds is a rough estimate of the probability of finding a hit by chance. A pre-trained computational predictor could rank the same compounds in silico from high probability of being a hit to low probability. We simulate the case where the chemist only selects the top 1% predictions for further wet lab testing, which is a reasonable cut off in real world high-throughput screens with very large compound libraries. By estimating the ratio of hits found in the top 1% subset that is actually tested in vitro, we then compute the folds of improvement as the ratio of the hit rates in each approach. Folds of improvement can be understood as the number of times that the experimental efficiency improves by using a predictor to filter unlikely hits and bring promising candidates to the top of the list.

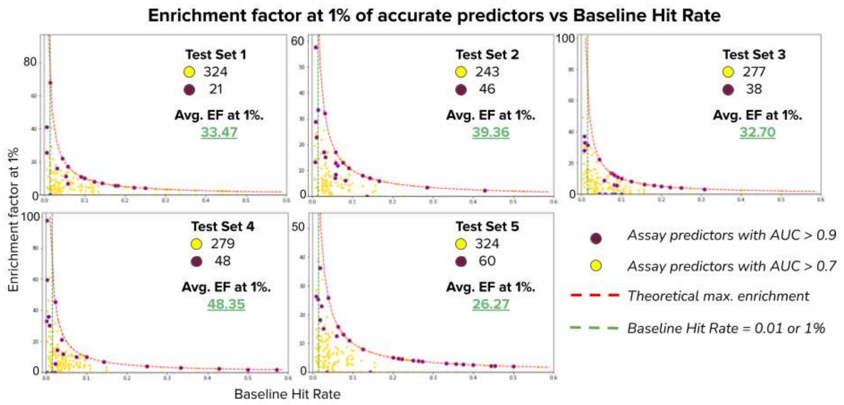

Supplementary Figure 7. Enrichment factor at 1% of accurate predictors vs Baseline Hit Rate. Each plot corresponds to the results in one split of the 5-fold cross-validation experiment (see Supplementary Figure 1). The points in the plots represent one assay predictor that uses one of the three data modalities (CS, GE or MO) or combinations of them. Assay predictors with AUROC > 0.7 are displayed in yellow and predictors with AUROC > 0.9 are displayed in purple. Assay predictors with AUROC < 0.7 are not shown. The horizontal axis represents the baseline hit rate, i.e., the proportion of compounds found to be hits in the set of tested compounds for an assay (see Supplementary Figure 6). The vertical axis presents the folds of improvement of assay predictions obtained with a machine learning predictor as a function of the baseline hit rate. Accurate predictors (AUROC > 0.9) often offer improvements up to the theoretical maximum (100% divided by the assay's baseline hit rate), and higher-fold improvements are only possible for assays with a lower baseline hit rate, i.e. with rare hits.

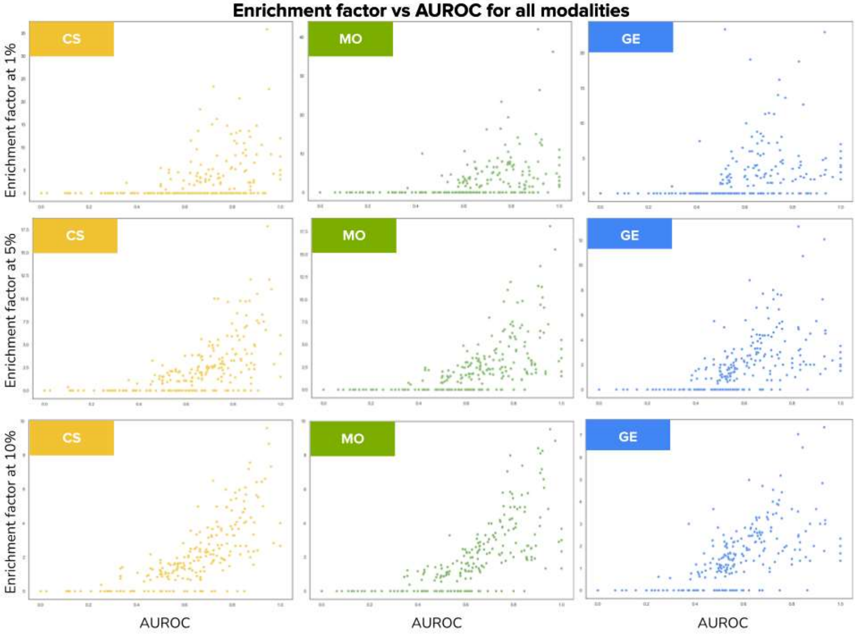

Supplementary Figure 8. Enrichment factor vs AUROC for all modalities at different thresholds. Plots in one column correspond to one modality, in a single row to one enrichment factor threshold. Points correspond to a performance for individual assay (270 in total).

Data fusion

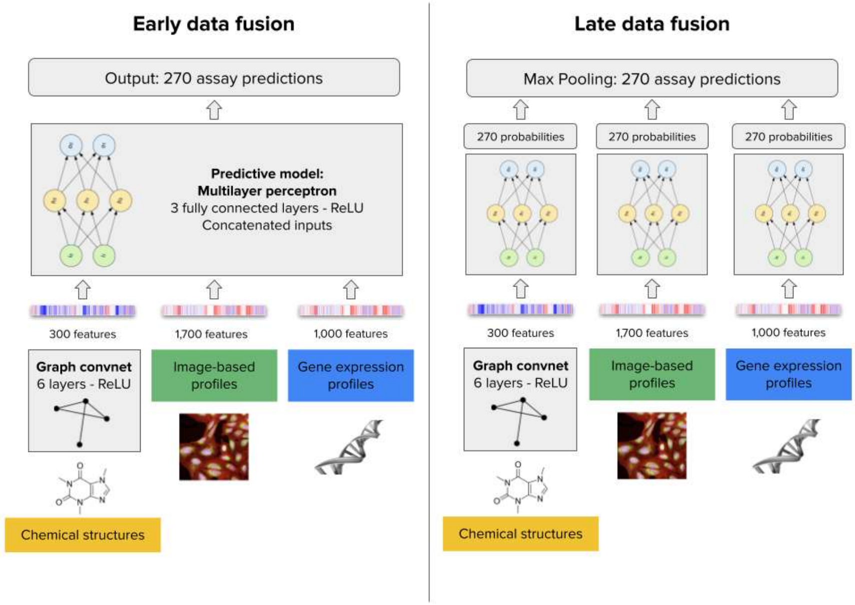

Supplementary Figure 9. Architecture of early and late data fusion models. The early data fusion model takes the three data modalities as input by obtaining features from each and then concatenating their representations. The architecture is a multilayer perceptron with three fully connected layers, 2,000 input features and 270 output predictions. The late data fusion model has one multilayer perceptron with three fully connected layers independently for each data modality. The three feature vectors are analyzed separately to produce 270 output probabilities in each case, which are later aggregated with a max-pooling operator to reduce them into a single vector of 270 assay predictions. The multilayer perceptron predictors in the early and late fusion approaches are all trained in a multi-task setting.

No fusion vs early fusion vs late fusion

Baseline: independent modalities (scaffold-based partitions)

	MO		GE		CS	
	Mean	Std	Mean	Std	Mean	Std
Mean AUPRC	0.252	0.021	0.234	0.038	0.232	0.036
Mean AUROC	0.637	0.021	0.592	0.034	0.630	0.018
AUC > 0.5	151.4	13.502	139.2	13.773	150.2	13.255
AUC > 0.7	83.2	11.100	57.2	16.316	88.4	6.066
AUC > 0.9	28.0	4.848	21.8	8.198	21.6	6.229

Early fusion — concatenation (scaffold-based partitions)

	GE-MO		MO-CS		GE-CS		GE-MO-CS	
	Mean	Std	Mean	Std	Mean	Std	Mean	Std
Mean AUPRC	0.214	0.045	0.251	0.021	0.219	0.028	0.221	0.021
Mean AUROC	0.586	0.038	0.632	0.031	0.577	0.061	0.582	0.038
AUC > 0.5	138.8	18.377	151.8	19.905	138.6	26.773	137.2	22.928
AUC > 0.7	59.2	12.215	87.8	15.531	63.4	21.663	59.8	14.516
AUC > 0.9	16.0	4.743	23.6	4.159	17.0	5.292	20.4	4.278

Late fusion — max pooling (scaffold-based partitions)

	GE-MO		MO-CS		GE-CS		GE-MO-CS	
	Mean	Std	Mean	Std	Mean	Std	Mean	Std
Mean AUPRC	0.261	0.026	0.267	0.034	0.251	0.039	0.265	0.032
Mean AUROC	0.652	0.028	0.661	0.027	0.645	0.026	0.665	0.031
AUC > 0.5	157.4	11.845	157.8	13.773	155.6	16.637	159.0	15.017
AUC > 0.7	86.0	9.670	98.8	7.430	87.0	9.566	96.4	10.877
AUC > 0.9	29.4	6.618	29.4	5.128	23.8	8.843	28.0	5.148

Supplementary Table 3. Mean performance of profiling modalities. Overall performance of profiling modalities and their combinations presented in the columns of the tables. Early fusion refers to concatenation of feature vectors before training predictive models, while late fusion refers to keeping the maximum prediction of individual models (see Supplementary Figure 8). The tables present four performance metrics in the rows: Mean AUPRC, mean AUROC, number of assays predicted with AUROC > 0.7, and number of assays predicted with AUROC > 0.9. For each experiment, we obtain the mean and standard deviation of the metric. In the case of the mean value for all metrics, higher numbers indicate better performance. Late fusion yields the largest number of predictors with AUROC > 0.9 overall, and also for all combinations of descriptors.

Data modalities

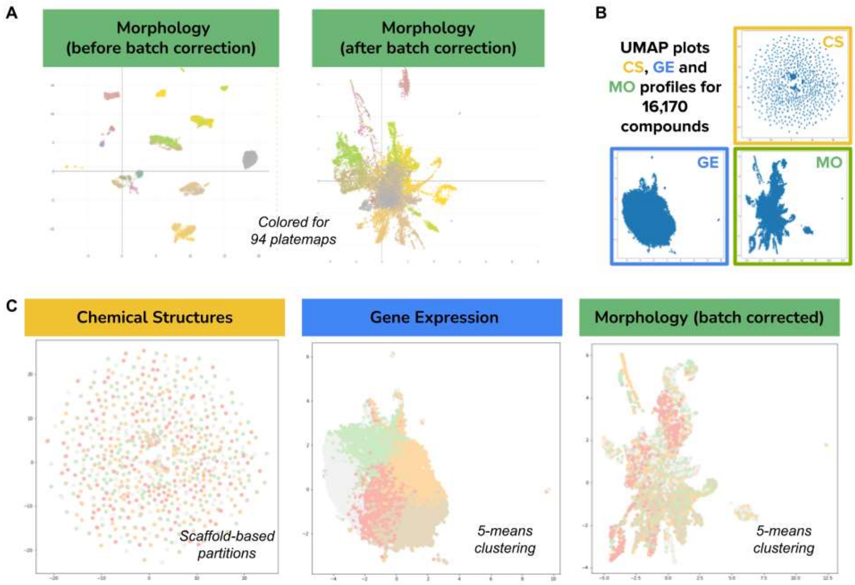

Supplementary Figure 10. Compound embeddings in three different feature spaces. Visualization of the high-dimensional feature vectors of all compounds using UMAP projections for the three data modalities used in this work. A) The morphology feature space originally was grouped by technical variation (plate maps), which was corrected using the Typical Variation Normalization (TVN) approach (see Methods) to report all experiments in the manuscript. The color palette for the 94 plate maps is continuous and may have similar tones for consecutive plates. B) Overview of the three feature spaces for all the 16,170 compounds included in the evaluation. Note that chemical structures (CS), gene expression (GE), and morphology (MO), all have very distinctive ways of organizing the signatures of compounds. While CS has many diverse small clusters, GE presents a single cloud, and MO has a central cloud with some medium clusters and branches. C) The same visualization as in B, but colored by clusters obtained for cross-validation experiments (see Supplementary Table 4). We partitioned each feature space using clustering to identify 5 groups for training and test splits. CS was split using Bemis-Murcko clustering, which is based on scaffold similarity, while the corresponding UMAP plot projects data points using the features of the full chemical structure (a different metric, which explains why the colors don't reveal scaffold clusters). GE and MO were split using k-means clustering, with k=5 for cross-validation in simulated control experiments to determine the influence of the data partition in the results (see Supplementary Table 4).

Cluster-based 5-fold cross validation

Scaffold-based splits — Real world setting

Average number of tested assays: **202.2**

	MO	MO-BC	GE	GE-S	CS-GC	CS-MF
Mean AUPRC	0.261	0.252	0.234	0.231	0.232	0.223
Mean AUROC	0.657	0.637	0.592	0.587	0.630	0.610
AUC > 0.5	160.0	151.4	139.2	138.8	150.2	146.8
AUC > 0.7	91.2	83.2	57.2	59.4	88.4	81.6
AUC > 0.9	27.0	28.0	21.8	18.4	21.6	21.0

Gene expression splits (simulation)

Average number of tested assays: **198.2**

	MO	MO-BC	GE	GE-S	CS-GC	CS-MF
Mean AUPRC	0.263	0.248	0.222	0.201	0.246	0.244
Mean AUROC	0.664	0.642	0.577	0.561	0.647	0.658
AUC > 0.5	155.6	150.2	127.6	127.2	153.2	157.4
AUC > 0.7	94.4	86.2	45.4	46.6	94.2	99
AUC > 0.9	27.4	23.6	14.2	12.6	22.6	22.4

Morphology(bc)-based splits (simulation)

Average number of tested assays: **186**

	MO	MO-BC	GE	GE-S	CS-GC	CS-MF
Mean AUPRC	0.224	0.207	0.199	0.198	0.225	0.245
Mean AUROC	0.634	0.600	0.562	0.564	0.631	0.652
AUC > 0.5	142	128.6	125.4	126.2	140.8	143.6
AUC > 0.7	72.8	63	49.2	49.2	81	82.6
AUC > 0.9	21.6	17	14.4	13.6	19.4	22.6

Random splits (simulation)

Average number of tested assays: **203**

	MO	MO-BC	GE	GE-S	CS-GC	CS-MF
Mean AUPRC	0.259	0.247	0.234	0.228	0.244	0.242
Mean AUROC	0.670	0.643	0.601	0.595	0.659	0.651
AUC > 0.5	163.6	154.2	145.6	144.0	157.6	157.8
AUC > 0.7	97.2	88.4	61.8	66.0	94.8	94.0
AUC > 0.9	26.2	22.0	20.4	17.4	25.8	23.4

Supplementary Table 4. Results of 5-fold cross-validation control experiments. The tables present the mean results of 5-fold cross-validation experiments according to different data partition policies (see Supplementary Figure 10). The scaffold-based splits reflect the real world scenario more closely, while other split policies are useful as control experiments to identify potential artifacts or biases in the data. For each data modality, we used two encoding versions as follows: MO: original features and batch corrected (BC) features. GE: original features and scaled (S) or renormalized features using the L1 norm. CS: graph convolutional (GC) features and Morgan fingerprints (MF). We use as baseline the results of scaffold-based splits, which are reported in the main text and were used to complete all the analysis in this work. Compared to scaffold-based splits, gene expression and random splits yield slightly higher mean AUROC for all other modalities, which confirms that separating training and test compounds randomly makes the prediction problem easier while not being fully informative in a real setting. Morphology splits decrease performance for all modalities, indicating that the k-means splitting by morphology features (see Supplementary Figure 10) disrupts effective learning by bringing together most compounds of certain assays into only one fold. This can be explained partially by the presence of technical artifacts and by real biological signal that could not be entirely separated with the adopted batch correction method. Finally, the difference in performance between graph convolutional representations of chemical structures and Morgan fingerprints is minor across all experiments. Graph convolutions (CS-GC) have slightly better performance in

the real world setting, and comparable performance in other splits. We used GC across all the reported experiments in the main manuscript.

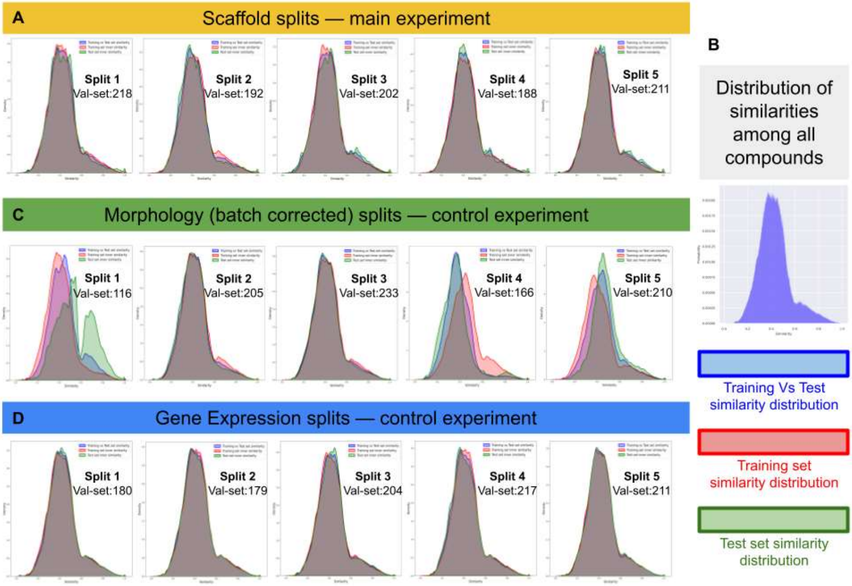

Supplementary Figure 11. Distribution of compound similarities across training-test splits. We computed the Tanimoto coefficient between Morgan fingerprints of all compounds in the dataset and obtained the distribution of scores (B), which indicates that most compounds are relatively equidistant to each other (consistent with Supplementary Figure 10C). After scaffold-based splitting, this distribution is preserved in training and test partitions in all five folds (A). No major distribution shift is observed with gene-expression splits (D), but two groups in the morphology splits (split 2 and 4) show larger differences likely explained by confounded signal between technical artifacts and biological effects (see Supplementary Table 4 and Supplementary Figure 10).

Assay data

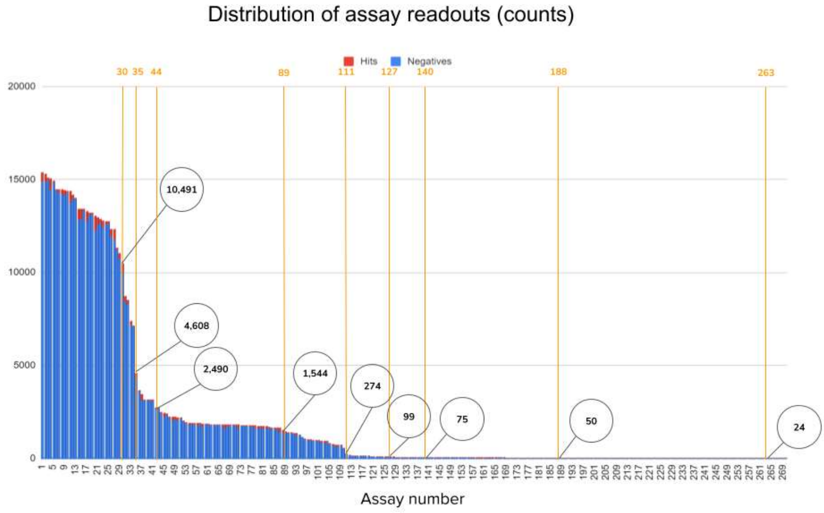

Supplementary Figure 12. Distribution of assay readouts. The plot shows in the horizontal axis assay identifiers sorted by readout count in decreasing order, and in the vertical axis the count of available readouts for each assay. Readouts can be positive hits (red) or negatives (blue). The circles in the plot indicate the readout count for specific assays in the distribution. Assay readouts follow a long tail distribution, with more than half of the assays having less than a few hundred readouts for training predictive models. Note that the ratio between hits and negative compounds is very small in general (average hit ratio 2.5%).

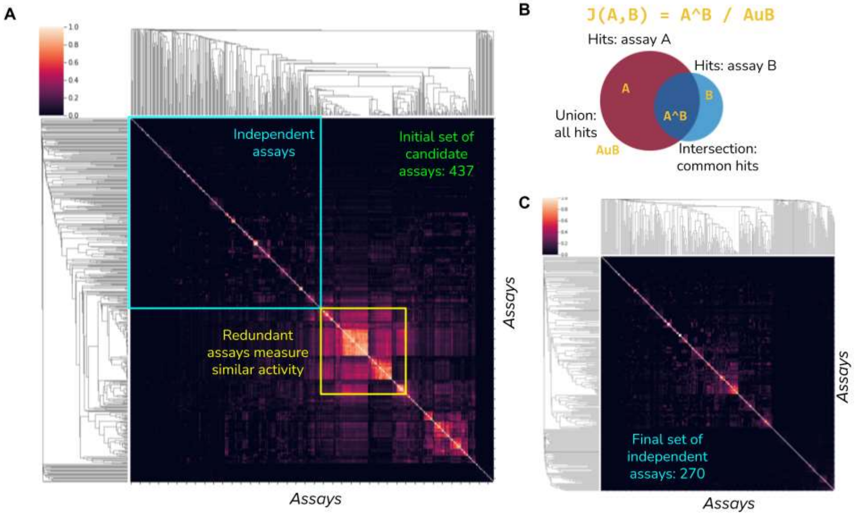

Supplementary Figure 13. Assay similarity. A) Matrix of assay similarities according to the Jaccard similarity between the sets of positive hit compounds. This matrix presents all the assays initially available for analysis (437). Groups of redundant assays were removed, defined as those with Jaccard index above 0.7 (for more details see Methods: Assay readouts). B) Illustration of the Jaccard similarity $J(A,B)$ between two assays A and B. Each assay has a set of positive hits and we compute the ratio of the intersection (hits in common) over the union (count of all total hits) as a metric of similarity between assays. Assays that have many hits in common are likely measuring the same biological activity, and were excluded from our analysis.

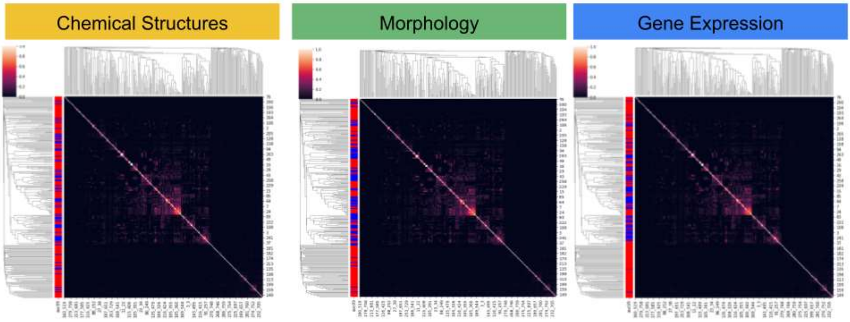

Supplementary Figure 14. Groups of assays predicted by each modality. The matrix of assay similarities is the same in the three cases: rows and columns are assays and the matrix values are the Jaccard index between the set of hits from two assays. The matrices are clustered in the rows and columns using hierarchical clustering to reveal groups of highly correlated assays. The only difference between the matrices is the coloring pattern of the left-hand side bar that indicates whether an assay is correctly predicted by the corresponding modality (chemical structures (CS), morphology (MO), and gene expression (GE)) in any of the cross-validation partitions (blue, red otherwise). This visualization is useful to reveal if the data modalities have preference for making better predictions with certain groups of assays that may have common biological activity. This result indicates that there are no major groups of activation, although accurate predictors tend to be close to each other in the cluster map. The dendrograms reveal a few assay clusters in the center of the matrices, and the visualization indicates that each modality tends to make accurate predictions in different groups; the accuracy patterns in the left of the matrices are different from modality to modality.

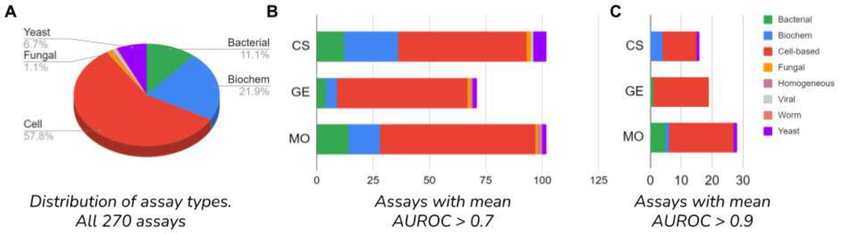

Supplementary Figure 15. Distribution of assay types as the performance threshold is decreased. The assays used in our study can be one of the seven types listed in the right hand side of the figure. A) Distribution of assays according to their type. B) Distribution of assays that can be predicted with a minimum accuracy of 0.7 AUROC by each of the three data modalities. C) Distribution of assays that can be predicted with a minimum accuracy of 0.9 AUROC by each of the three data modalities. These distributions show that none of the modalities has a strong preference for one type of assay, and that they can predict a diverse array of biological activity.

A

0.9 median AUROC	CS	GE	MO	CS+GE	CS+MO	GE+MO	CS+GE+MO	Evaluated assays
Cell-based	7.05%	11.54%	13.46%	10.90%	16.03%	17.31%	16.67%	156
Biochemical	6.78%	0.00%	1.69%	1.69%	3.39%	0.00%	1.69%	59
Bacterial	0.00%	3.33%	16.67%	0.00%	6.67%	3.33%	3.33%	30
Yeast	5.56%	0.00%	5.56%	0.00%	11.11%	0.00%	0.00%	18
Fungal	0.00%	0.00%	0.00%	0.00%	0.00%	0.00%	0.00%	3
Viral	0.00%	0.00%	0.00%	0.00%	0.00%	0.00%	0.00%	2
Worm	0.00%	0.00%	0.00%	0.00%	0.00%	0.00%	0.00%	1
Homogeneous	0.00%	0.00%	0.00%	0.00%	0.00%	0.00%	0.00%	1

B

0.7 median AUROC	CS	GE	MO	CS+GE	CS+MO	GE+MO	CS+GE+MO	Evaluated assays
Cell-based	36.54%	37.18%	44.23%	47.44%	46.15%	51.28%	50.00%	156
Biochemical	40.68%	8.47%	23.73%	32.20%	42.37%	18.64%	33.90%	59
Bacterial	40.00%	13.33%	46.67%	23.33%	56.67%	36.67%	43.33%	30
Yeast	33.33%	11.11%	11.11%	33.33%	33.33%	16.67%	16.67%	18
Fungal	66.67%	33.33%	33.33%	33.33%	66.67%	33.33%	33.33%	3
Viral	50.00%	0.00%	0.00%	50.00%	50.00%	0.00%	50.00%	2
Worm	0.00%	100.00%	100.00%	100.00%	0.00%	100.00%	0.00%	1
Homogeneous	0.00%	0.00%	100.00%	0.00%	100.00%	100.00%	100.00%	1

Supplementary Table 5. Predicted assays by type at the performance thresholds. A) Percentage of assays (out of 270 evaluated) that can be predicted by one modality or their combinations (columns) at high accuracy (>0.9 AUROC) grouped by assay type (rows). B) Same information as A but with an accuracy threshold of 0.7.
