# Supplementary material for: Predicting compound activity from phenotypic profiles and chemical structures
Source: Nat Commun. 2023 Apr 8;14:1967. doi: 10.1038/s41467-023-37570-1 (PMC10082762; doi:10.1038/s41467-023-37570-1)
Supplement: Supplementary file 2 — Reporting Summary [file 41467_2023_37570_MOESM2_ESM.pdf]

nature portfolio

Corresponding author(s): Juan C. Caicedo

Last updated by author(s): Mar 15, 2023

# Reporting Summary

Nature Portfolio wishes to improve the reproducibility of the work that we publish. This form provides structure for consistency and transparency in reporting. For further information on Nature Portfolio policies, see our Editorial Policies and the Editorial Policy Checklist.

## Statistics

For all statistical analyses, confirm that the following items are present in the figure legend, table legend, main text, or Methods section.

| n/a | Confirmed | |
|---|---|---|
| [ ] | [x] | The exact sample size (*n*) for each experimental group/condition, given as a discrete number and unit of measurement |
| [ ] | [x] | A statement on whether measurements were taken from distinct samples or whether the same sample was measured repeatedly |
| [x] | [ ] | The statistical test(s) used AND whether they are one- or two-sided<br>*Only common tests should be described solely by name; describe more complex techniques in the Methods section.* |
| [x] | [ ] | A description of all covariates tested |
| [x] | [ ] | A description of any assumptions or corrections, such as tests of normality and adjustment for multiple comparisons |
| [ ] | [x] | A full description of the statistical parameters including central tendency (e.g. means) or other basic estimates (e.g. regression coefficient) AND variation (e.g. standard deviation) or associated estimates of uncertainty (e.g. confidence intervals) |
| [x] | [ ] | For null hypothesis testing, the test statistic (e.g. *F*, *t*, *r*) with confidence intervals, effect sizes, degrees of freedom and *P* value noted<br>*Give P values as exact values whenever suitable.* |
| [x] | [ ] | For Bayesian analysis, information on the choice of priors and Markov chain Monte Carlo settings |
| [x] | [ ] | For hierarchical and complex designs, identification of the appropriate level for tests and full reporting of outcomes |
| [x] | [ ] | Estimates of effect sizes (e.g. Cohen's *d*, Pearson's *r*), indicating how they were calculated |

*Our web collection on statistics for biologists contains articles on many of the points above.*

## Software and code

Policy information about availability of computer code

| | |
|---|---|
| Data collection | No software was used |
| Data analysis | The ChemProp software was used for training machine learning models and can be found on GitHub https://github.com/chemprop/chemprop (version from 2021, commit hash 93e0ae).<br>For data filtering and calculation of Morgan fingerprints the RDKit v2021.09.4 was used.<br>The analysis code to reproduce the experiments reported in the paper can be found in the following link: https://github.com/carpenterlab/puma_project with DOI https://doi.org/10.5281/zenodo.7742610<br>All of those above packages and code operated with Python3.7+. |

For manuscripts utilizing custom algorithms or software that are central to the research but not yet described in published literature, software must be made available to editors and reviewers. We strongly encourage code deposition in a community repository (e.g. GitHub). See the Nature Portfolio guidelines for submitting code & software for further information.

## Data

Policy information about availability of data

All manuscripts must include a data availability statement. This statement should provide the following information, where applicable:

- Accession codes, unique identifiers, or web links for publicly available datasets
- A description of any restrictions on data availability
- For clinical datasets or third party data, please ensure that the statement adheres to our policy

The morphological and gene-expression profiles were originally created and published by Wawer, M. J. et al., and can be downloaded from: http://www.broadinstitute.org/mlpcn/data/Broad.PNAS2014.ProfilingData.zip

The Cell Painting images were made available by Bray et al., and can be obtained from the following link: http://gigadb.org/dataset/100351. Also available on the Image Data Resource (IDR) under accession number idr0016 and on the Cell Painting Gallery of AWS Open Data at s3://cellpainting-gallery/cpg0012-wawer-bioactivecompoundprofiling/. The subsets of gene expression profiles and morphological profiles used in this study are available on Zenodo: https://doi.org/10.5281/zenodo.7729583

The assay data to reproduce the analysis in the paper is available in the project GitHub repository: https://github.com/carpenterlab/puma_project/tree/main/data and Zenodo: https://doi.org/10.5281/zenodo.7729583

## Human research participants

Policy information about studies involving human research participants and Sex and Gender in Research.

| | |
|---|---|
| Reporting on sex and gender | NA |
| Population characteristics | NA |
| Recruitment | NA |
| Ethics oversight | NA |

Note that full information on the approval of the study protocol must also be provided in the manuscript.

# Field-specific reporting

Please select the one below that is the best fit for your research. If you are not sure, read the appropriate sections before making your selection.

- [x] Life sciences
- [ ] Behavioural & social sciences
- [ ] Ecological, evolutionary & environmental sciences

For a reference copy of the document with all sections, see nature.com/documents/nr-reporting-summary-flat.pdf

# Life sciences study design

All studies must disclose on these points even when the disclosure is negative.

| | |
|---|---|
| Sample size | No data collection was performed in this study. No statistical methods were used to predetermine the sample size. Final dataset after filtering consists of 270 assays and 16,170 compounds. |
| Data exclusions | The total number of compounds in the library that had the three types of information required to conduct the analysis in our project (Cell Painting images, L1000 profiles, and assay readouts) was 16,978. We applied all pan-assay interference (PAINS) filters 49 implemented in RDKit, which removed 786 compounds, resulting in 16,210 compounds. Next, we removed all assays without hits reducing the set of candidate assays from 496 to 437. Then, we calculated the Jaccard score between assay hits to identify redundant assays, i.e., assays that measure similar activity resulting in the same hits. The Jaccard similarity matrix (437x437) was thresholded at 0.7 to remove highly redundant assays, and hierarchical clustering with the cosine distance metric was applied for determining further groups of redundant assays. Finally, we removed frequent hitters, defined as compounds that are positive hits in at least 10% of the assays (by being hits in 30 assays or more) and an additional step of removing assays that remain without any hit. In the end, the final dataset consists of 16,170 compounds and 270 assays. |
| Replication | We did not perform biological or technical replication in this study as all data was created in the previous studies. For the computational analysis of machine learning algorithms, we performed cross validation for all settings of training experiments (see Training / Test splits subsection in the Methods). |
| Randomization | To allocate compounds to experimental groups, compounds were grouped into scaffolds. Scaffolds were randomly split into training and test sets for model training (main result, spitting by scaffolds). We allocated samples by similarity of morphological or gene expression profiles using clustering, which is not random because this allocation groups similar compounds together. The scaffold-based approach is considered to be closer to a real-world practical scenario (for predicting properties of novel compounds). We conducted an additional experiment by |

| | |
|---|---|
| | sampling compounds at random for five-fold cross-validation together with the corresponding random holdout test sets (random ~20% of compounds, 10 repetitions). This experiment is also reported as a baseline. |
| Blinding | There was no blinding performed in this study because the samples were not evaluated by human experts. Instead, we follow a cross-validation approach for performance evaluation. |

# Reporting for specific materials, systems and methods

We require information from authors about some types of materials, experimental systems and methods used in many studies. Here, indicate whether each material, system or method listed is relevant to your study. If you are not sure if a list item applies to your research, read the appropriate section before selecting a response.

## Materials & experimental systems

| n/a | Involved in the study |
|---|---|
| ☒ | ☐ Antibodies |
| ☒ | ☐ Eukaryotic cell lines |
| ☒ | ☐ Palaeontology and archaeology |
| ☒ | ☐ Animals and other organisms |
| ☒ | ☐ Clinical data |
| ☒ | ☐ Dual use research of concern |

## Methods

| n/a | Involved in the study |
|---|---|
| ☒ | ☐ ChIP-seq |
| ☒ | ☐ Flow cytometry |
| ☒ | ☐ MRI-based neuroimaging |
